# Supplementary material for: COVID-19 pandemic hits differently: examining its consequences for women’s livelihoods and healthcare access – a cross-sectional study in Kinshasa DRC
Source: BMJ Open. 2023 Sep 7;13(9):e072869. doi: 10.1136/bmjopen-2023-072869 (PMC10496687; doi:10.1136/bmjopen-2023-072869)
Supplement: Author's manuscript [file bmjopen-2023-072869.draft_revisions.pdf]

BMJ Open is committed to open peer review. As part of this commitment we make the peer review history of every article we publish publicly available.

When an article is published we post the peer reviewers' comments and the authors' responses online. We also post the versions of the paper that were used during peer review. These are the versions that the peer review comments apply to.

The versions of the paper that follow are the versions that were submitted during the peer review process. They are not the versions of record or the final published versions. They should not be cited or distributed as the published version of this manuscript.

BMJ Open is an open access journal and the full, final, typeset and author-corrected version of record of the manuscript is available on our site with no access controls, subscription charges or pay-per-view fees (http://bmjopen.bmj.com).

If you have any questions on BMJ Open's open peer review process please email info.bmjopen@bmj.com

BMJ Open

The COVID-19 Pandemic Hits Differently: Its Consequences on Congolese Women’s Livelihood and Family Planning Utilization

Journal:	*BMJ Open*
Manuscript ID	bmjopen-2023-072869
Article Type:	Original research
Date Submitted by the Author:	18-Feb-2023
Complete List of Authors:	Nkweleko Fankam, Falone; Dalarna University School of Health and Welfare,<br>Ugarte, Wiliam; Uppsala University, Department of Women's and Children's Health<br>Akilimali, Pierre; University of Kinshasa Faculty of Sciences, Department of Biostatistics and Epidemiology, Kinshasa School of Public Health<br>Ewane, Etah Junior; University of Buea, Department of Microbiology and Parasitology, Faculty of Science,<br>Eva, Åkerman; Karolinska Institute, Department of Women's and Children's Health
Keywords:	COVID-19, Public health < INFECTIOUS DISEASES, Reproductive medicine < GYNAECOLOGY

SCHOLARONE™
Manuscripts

I, the Submitting Author has the right to grant and does grant on behalf of all authors of the Work (as defined in the below author licence), an exclusive licence and/or a non-exclusive licence for contributions from authors who are: i) UK Crown employees; ii) where BMJ has agreed a CC-BY licence shall apply, and/or iii) in accordance with the terms applicable for US Federal Government officers or employees acting as part of their official duties; on a worldwide, perpetual, irrevocable, royalty-free basis to BMJ Publishing Group Ltd ("BMJ") its licensees and where the relevant Journal is co-owned by BMJ to the co-owners of the Journal, to publish the Work in this journal and any other BMJ products and to exploit all rights, as set out in our licence.

The Submitting Author accepts and understands that any supply made under these terms is made by BMJ to the Submitting Author unless you are acting as an employee on behalf of your employer or a postgraduate student of an affiliated institution which is paying any applicable article publishing charge ("APC") for Open Access articles. Where the Submitting Author wishes to make the Work available on an Open Access basis (and intends to pay the relevant APC), the terms of reuse of such Open Access shall be governed by a Creative Commons licence – details of these licences and which Creative Commons licence will apply to this Work are set out in our licence referred to above.

Other than as permitted in any relevant BMJ Author's Self Archiving Policies, I confirm this Work has not been accepted for publication elsewhere, is not being considered for publication elsewhere and does not duplicate material already published. I confirm all authors consent to publication of this Work and authorise the granting of this licence.

For peer review only

The COVID-19 Pandemic Hits Differently: Its Consequences on Congolese Women's Livelihood and Family Planning Utilization

Nkweleko Fankam Falone[1], William J. Ugarte[2], Pierre Akilimali Z[3], Ewane Etah Junior[4], Eva Åkerman[5]

Authors Affiliation:

1. School of Health and Wealth fare, Dalarna University, Falun, Sweden.
2. Department of Women's and Children's Health, Uppsala University, Uppsala, Sweden.
3. Department of Biostatistics and Epidemiology, Kinshasa School of Public Health, University of Kinshasa, Kinshasa P.O. Box 11850, the Democratic Republic of the Congo
4. Department of Microbiology and Parasitology, Faculty of Science, University of Buea, P.O.Box 63, Buea, SWR, Cameroon
5. Department of Women's and Children's Health, Karolinska Institute, Stockholm, Sweden.

Corresponding authors: eva.akerman.2@ki.se, fankam93@yahoo.com

Disclaimers

All the views and opinions presented in this paper are that of the authors and do not represent that of any institution

For peer review only

Abstract

Objectives: The emergence of the COVID -19 pandemic came with multiple preventive actions as primary intervention to contain the spread of the virus. Globally, countries are facing enormous challenges with social, economic and health service utilization consequences. Democratic Republic of Congo (DRC) was one of the African countries to implement strict lockdown during the early days of the outbreak, this resulted in shortages and access to services. The adverse related shocks of the pandemic left the country with unpleasant consequences. This study aimed to investigate the association between the COVID-19 pandemic-related factors, sociodemographic factors, and the need for visiting health facilities including family planning services in women aged 15-49 years in the DRC.

Methods: We conducted a secondary analysis of performance monitoring for action (PMA) cross-sectional COVID-19 phone survey in Kinshasa DRC. 1325 women aged 15-49 from the Kinshasa-province participated in the survey. Bivariate and multivariate logistic regression was used to assess associations.

Results: The COVID-19 pandemic and related factors affected 92% of women in the Kinshasa-province socioeconomically. Majority witnessed high levels of economic dependability on their partners/other sources for their basic needs to be meet, and many more worried about the future impact of the pandemic on their household-finances. Over 50% of women did not attempt visiting a health-service and among the top reasons was the fear of being infected with COVID-19 and not being able to afford services. We found significant association of women's age group and contraceptive use. The need/use of contraceptive was higher among women aged 25-34 years than those aged 35-49 and 15-24.

Conclusion: Effective social/economic support for women and girls during pandemic and in crisis settings is essential as this has lasting and beneficial effects on many domains of their lives including their ability to access health services and contraceptives of their choice.

For peer review only

Data Availability statement

Datasets used for the study are not publicly available and are managed very restrictively according to the ethics approval and data usage guidelines by PMA. The request for the data set should be made through the PMA data request system.

Summary Box

- **What is already known on this topic**?
  - The COVID-19 pandemic has affected the proper access and delivery of essential and quality health care services.
  - Several findings have shown the effects of the COVID-19 pandemic related lockdown on the entire population.

- **What this study adds** - *summarise what we now know as a result of this study that we did not know before*
  - Women are bearing the brunt of the economic and societal fallouts of the pandemic.
  - Women faced high levels of economic reliant on their partners during the COVID -19 period than before.
  - Many women not attempting to visit health services but still do not want to get pregnant because of the pandemic.

- **How this study might affect research, practice or policy** - *summarise the implications of this study*
  - Alternative means for women to be able to get the sexual and reproductive health services of their choice during pandemic period should be included in rapid intervention strategies as well as interventions for economic empowerment.

For peer review only

Introduction

The COVID-19 pandemic resulted in multiple restrictive actions as a primary preventive intervention in most countries[1]. Globally, countries were remarkably unprepared with many more still far from ready to cope with the huge increase in critically ill persons. A scoping review including studies from both high income and low-middle income countries by Bolarinwa *et al*[2] reported the severe impact of COVID-19 on family planning (FP) services, sexual behavior, and maternal and child health services because of limited access to preferred choices, especially for those with FP needs. Further, the impact of the pandemic means that, globally, such sexual and reproductive health (SRH) services and support ranging from condoms, contraceptives, HIV testing and treatment, and uninterrupted hormone treatment for transgender persons might be limited if available at all[3]. Consequently, the pandemic has been a threat to several countries posing severe stretch to health systems, especially in those with already preexisting crises and humanitarian conditions like the Democratic Republic of the Congo (DRC)[4]. Thus, COVID-19 intensifies pre-existing health hardships, for instance, the DRC had been experiencing several diseases occurrence in the last two years such as Measles, Cholera, and Ebola outbreaks[4]. The Ebola outbreak is considered the second largest Ebola outbreak in the world[5] which claimed the lives of thousands of inhabitants 6[5]. The long-lasting Ebola epidemic combined with insecurity and armed conflicts caused an inefficient and insufficient supply of SRH services, particularly for women and young people[6].In addition, lockdowns and resources allocated to the COVID-19 response further compounded the problem by limiting the access to essential SRH services for women and girls[6].

Furthermore, the early lockdown after discovering the first case on 10 March 2020, in Kinshasa included a suspension of flights from COVID-19–infected countries, movements prohibition within the country further limiting access to service,[7] and possibly further hindering the supply chain and increased shortages for those who could access services. Also, people were further frustrated and devasted by

For peer review only

severe social and economic hurdles, which demanded intensified struggles for women and girls[8]. A recent publication showed an increased need for contraception among nulliparous women in the DRC during the COVID-19 pandemic[9]. Such demand was not observed among women already having children.

The necessity to set aside resources and provide equal access to essential health care services including FP services in crisis/pandemic settings is critical. Various health and government institutions have been promoting different measures to contain the pandemic that focus on individual behaviors with little attention to the social, economic, and contextual factors[10] as well as the psychological factors[11,12]. This research was hence necessary to better understand the social, and economic consequences of the pandemic on the need for health services including FP among women, specifically those in the urban setting of the DRC context. Recognizing these factors and consequences would potentially help stakeholders better strengthen their preparation, budgeting, and delivery of SRH services globally considering the present context and have a better grasp of possible short- and long-term consequences of lockdown on women.

The study aimed to investigate if there is an association between the COVID-19 pandemic-related factors, sociodemographic factors, and the need for visiting health facilities including family planning services in women aged 15-49 years in the DRC.

Methods

Study Design, Population, and Sample size

The study aim was assessed using a cross-sectional design and was conducted by phone between May and June 2020. Participants were women aged 15-49 years randomly selected from households in Kinshasa province, who participated in the Performance Monitoring for Action (PMA) baseline survey between December 2019 and February [12]. Overall, 69.5% of the baseline participants consented to take part in future studies and provided phone numbers. Of the 1773 total eligible participants, 77.6%

For peer review only

(n=1375) were reached, finally, 96.3% (n=1325) completed the phone survey for a final response rate of 74.7% among eligible women[13]. Stata survey procedures were used to account for the sampling design and selection weights.

Patient and Public Involvement

This study involves data gotten from PMI COVID-19 phone survey in Kinshasa. There was no patient or public involvement.

Data Collection

The survey was conducted by trained women in the designated localities using Open Data Kit (ODK). This is a publicly available software application to collect and administer data using mobile phones. The ODK questionnaire was adapted to give spontaneous skip restrictions to reduce data entry errors[14]. The instant aggregation of data allows for concurrent processing and course corrections while still in the field. During the data collection stage, there was regular monitoring and feedback given to interviewers in the event of errors, missing data, or form submissions problems on the central server.

Measurements

The outcome variables for the analysis constituted the need to access health services including family planning and the current use of contraceptives. These outcome variables were considered as: the need for visiting health services, difficulties in accessing health services including FP services, current contraceptive use at the time of data collection, and behavioural change in demand for contraceptive use. Each category had its representing value[15]

Accessing health services in times of COVID-19 as the exposure variable comprised seven closed ended multiple choice questions with a single response option considered: a) unable to access services because of government restrictions on movement, b) unable to afford FP services, c) fear of being infected with

For peer review only

COVID-19 at healthcare facilities, d) did not attempt to access healthcare services or did not experience, e) difficulties accessing car/ no transportation to access healthcare services, f) healthcare facility or doctor's office closed, appointment not possible, g) preferred contraceptive method unavailable

The socio demographic variables included age, education, place of residence, pregnancy status, marital status, economic status, and decision-making ability on household purchases. Economic status was based on the ability of respondents to take care of their needs and those of their families or are dependent on someone for those needs to be met. This was coded by PMA as being reliant on husband/partner, respondent themselves, respondent and husband/partner, or someone else and no response.

Data Analysis

Statistical analysis was done using SPSS version 26 (IBM Corp., Armonk, NY). Descriptive statistics included mean and standard deviation for quantitative variables and absolute frequencies and percentages for qualitative ones. Data were checked for normality and even distribution. Pearson's Chi-square (χ^2) test was used to compare differences in proportions between groups. Both bivariate and multivariate logistic regression analyses were used. The independent variables associated with the outcomes at *p* value < 0.20 in the bivariate analysis were included in the multivariable analysis. The multivariate analysis was adopted for potential confounders. Logistic regression models were performed on the two dependent variables: the need to access healthcare services and the current use of contraceptives. During bivariate analysis, the independent variables associated with the outcomes at *p* value < 0.20 were included in the multivariable analysis. The variance inflation factor (VIF) was used to test for multicollinearity between variables (predictors) in the regression model. All statistical analyses were considered significant at $p < 0.05$.

For peer review only

Results

Table 1 presents the sociodemographic characteristics of the women included in the study. A total of 1325 women consented to participate in the study with a mean age of 29.49 ± 8.97 years. Most of the women were well-educated corresponding with 66.8% secondary and about one-third (29.9%) tertiary education. More than half (53.0%) of the women were unmarried. At the time the time of data collection, the majority (94.0%) of the women were not pregnant.

Table 1: Demographic characteristics of the study population, N = 1325

**Characteristics**	**Frequency (n)**	**Percentage (%)**
**Age group (years)**		
15 – 24 (Youth)	474	35.8
25 – 34 (Young Adult)	464	35.0
35 – 49 (Adults)	387	29.2
**Total**	**1325**	**100**
**Mean age (years)**	**29.49 ± 8.97**	
**Educational level**		
Primary	44	3.3
Secondary	884	66.8
Tertiary	395	29.9
**Total**	**1323[a]**	**100**
**Marital status**		
Married or cohabiting	620	47.0
Unmarried	703	53.0
**Total**	**1323[a]**	**100**
**Currently Pregnant**		
No	1246	94.0
Yes	72	5.4

For peer review only

Unsure/ No response	7	0.6
**Total**	**1325**	**100**

[a] ***Missing value excluded***

Table 2 depicts the socio-economic impact of COVID-19 restrictions on women in Kinshasa province. Nearly all the women experienced household income loss either partially (33.0%) or completely (59.0%). Overall, 1214 (92.0%) of women experienced household income loss due to COVID-19. Similarly, most (89.8%) of the women were worried about the impact of COVID-19 on their future household finances. In terms of food security, 36.8% of the women affirmed to have witnessed their household members starved the whole day and night because there was not enough food to eat. Equally, the women were asked questions on "whether they were currently economically reliant on their husbands/partners for basic needs" and, "whether they were more economically reliant on husband/partner now than before COVID-19 restrictions began". More than a half 50% of the women didn't respond to these questions. Of 612 women that responded to the former question, 392 (64.1%) of them affirmed that they were currently economically reliant on their husbands/partners for basic needs. In addition, 289 (74.0%) of the 391 women agreed to have been more economically reliant on their husband/partners now than before COVID-19 restrictions began as shown in Table 2.

Table 2: Socio-economic impact of COVID-19 restrictions on women in Kinshasa province

**Characteristics**	**Impact due to COVID**	
	**Frequency (n)**	**Percentage (%)**
**Household income loss**		
None	107	8.0
Partial	438	33.0
Complete	776	59.0
**Total**	**1321[a]**	**100**
**Worried about the impact of COVID-19 on future household finances**		
Yes	1177	89.8
No	133	10.2

For peer review only

**Total**	**1310[a]**	**100**
**Lack of food for 24 hours**		
Yes	486	36.8
No	834	63.2
**Total**	**1320[a]**	**100**
**Frequency of food inadequacy (n=486)**		
Rarely (1 – 2 times)	171	35.2
Sometimes (3-10 times)	239	49.2
Often (> 10 times)	75	15.4
Don't know	01	0.2
**Total**	**486**	**100**
**Currently economically reliant on husband/partner for basic needs**		
Yes	392	29.6
No	220	16.6
No response	713	53.8
**Total**	**1325**	**100**
**More economically reliant on husband/partner now than before COVID-19 restrictions began**		
Yes	289	21.8
No	102	7.7
No response/ do not know	934	70.5
**Total**	**1325**	**100**

Missing values excluded

With regards to women's need to visit healthcare facilities for seeking services including family planning during the COVID-19 restrictions, only 402 (30.0%) needed healthcare services, of those, 325 (81.0%) successfully visited the facilities and had access to healthcare. Furthermore, the different reasons for accessing healthcare were determined, ranked, and presented in Figure 1. Most (43.0%) of the women visited the health facilities purposely for general healthcare services while other women went to the health facilities due to their child's health (14.0%), other personal reasons (11.0%), family planning services (7.0%), antenatal care (6.0%).

For peer review only

Equally, the reasons for not accessing healthcare services among the women were evaluated and depicted in Figure 2. More than a half (58%) of the women reported not attempting to access healthcare services or did not experience difficulties in accessing care. However, some of the women did not access healthcare because of fear of being infected with COVID-19 (14.0%) while others were unable to afford healthcare services (12.0%). Also, some possible reasons included: no transportation to access healthcare (6%), government restriction on movement (4%), healthcare facilities or doctors' offices closed (2%), and partner disapproval (1%).

There was a self-reported attitudinal change in the need for family planning due to the COVID-19 pandemic among married or cohabiting and unmarried women as shown in Table 3. A total of 111 women affirmed that they would change their minds about getting pregnant. Though not statistically significant between unmarried and married women. Equally, most unmarried women (57.6%) responded that they would be very unhappy if gotten pregnant as opposed to married women (26.9%).

Furthermore, a significant proportion of the unmarried women (62.0%) accepted that they would wait for many years before putting to birth as compared to just a few married women (34.7%). Similarly, most of the unmarried women (61.0%) concurred that they were currently using any method to delay or avoid pregnancy relative to the married women (38.7%) as presented in Table 3

For peer review only

Table 3: Attitudinal changes due to COVID-19 restrictions between married/cohabiting compared to unmarried women

Characteristics	Categories	Responses		P value
		Married or cohabiting n = 620 frequency (%)	Un-married n = 703 frequency (%)	
**Change of mind about being pregnant due to COVID-19**	Yes (n =111)	50 (8.0)	61 (8.7)	0.691
**Would you prefer not to have any more children?**	No more children (n=193)	137 (22.1)	56 (8.0)	**< 0.001***
**Respondents' feelings if pregnant**	Very unhappy (n=572)	167 (26.9)	405 (57.6)	**< 0.001***
**Currently, using any method to delay or avoid getting pregnant?**	No (n=669)	240 (38.7)	429 (61.0)	**< 0.001***
**Waiting time to put to birth**	Years (n=651)	215 (34.7)	436 (62.0)	**< 0.001***

For analytical purposes, Married women constituted Legally married and those living with partners (Cohabiting); freq = Frequency

For peer review only - http://bmjopen.bmj.com/site/about/guidelines.xhtml

Both bivariate and multivariate analyses were conducted to assess the relationship between the current use of contraceptives and socio-demographic factors Table 4. We observed that women aged 25 – 34 years (50.4%) were significantly more using contraceptives than those aged 35 – 49 years (42.0%) and 15 – 24 years (37.1%). A chi square test performed showed a significant association between women's age groups and current use of contraceptives. More so, the current use of contraceptives increased with a decrease in educational level. In other words, most women with tertiary education were currently using contraceptives more than those in secondary education (41.0%) and primary education (40.9%). With regards to marital status, more married women (51.3%) were significantly using contraceptives than unmarried women (36.1%).

Though not significant, women who experienced household income loss (43.7%) used contraceptives more than those who didn't experience household income loss (39.3%). Similarly, women who were economically reliant on their husbands/partners (51.5%) were reported of currently using contraceptives than those not experiencing economic reliance (50.9%). After controlling for confounders in the multivariate logistic analyses, marital status (specifically those married) was significantly associated (AOR: 2.04 (95% CI: 1.56 – 2.67, $p < 0.001$) with the current use of contraceptives. Equally, women with secondary education (AOR: 0.72 (95% CI: 0.57 – 0.92, $p = 0.009$) had a protective role in the current use of contraceptives.

For peer review only

Table 4: Bivariate and multivariate logistic regression analysis on the current use of contraceptives and socio-demographic factors

Variables	Bivariate analysis		Multivariate analysis (n = 572)		
	COR (95% CI)	*P-value*	AOR (95% CI)	*P-value*	*VIF*
**Age groups**					**1.039**
15 – 24 (Youth)	**Ref**		**Ref**		
25 – 34 (Young Adult)	1.72 (1.34 – 2.24)	**< 0.001***	1.22 (0.91 – 1.64)	**0.177**	
35 – 49 (Adults)	1.23 (0.93 – 1.61)	**0.149**	0.78 (0.56 – 1.08)	**0.136**	
**Educational level**					**1.014**
Tertiary	**Ref**		**Ref**		
Secondary	0.74 (0.58 – 0.94)	**0.014***	0.72 (0.57 – 0.92)	**0.009***	
Primary	0.73 (0.39 – 1.39)	**0.350**	0.70 (0.37 – 1.35)	**0.295**	
**Marital status**					**1.000**
Unmarried	**Ref**		**Ref**		
Married/cohabiting	1.86 (1.49 – 2.32)	**<0.001***	2.04 (1.56 – 2.67)	**<0.001***	
**Economic reliance**					**1.052**
Not reliant	**Ref**		**Ref**		
Reliant	1.03 (0.74 – 1.43)	**0.883**	-	**-**	
**Household income Loss**					**1.001**
No	**Ref**		**Ref**		
Yes	1.20 (0.80 – 1.80)	**0.379**	-	**-**	

Where, Ref = Reference group; COR = Crude Odds Ratio; AOR: Adjusted Odds Ratio

*** Significant factor (p <0.05) VIF: Variance Inflation factor**

To ascertain the association between sociodemographic factors and the need to access healthcare including family planning, both bivariate and multivariate analyses were performed Table 5. The bivariate analysis showed that, those aged between 25–34 years (COR = 1.60 (95% CI:1.06 – 1.83), those in secondary education (COR = 0.77 (95% CI: 0.06 – 1.00), married women (COR = 1.74 (95% CI:1.38- 2.21) and household income loss (COR = 1.39 (95% CI:0.88 – 2.20) were associated with the need to access healthcare services. However, after using the multivariable analysis, we observed that, women aged 25 – 34 years (AOR = 1.76, 95% CI: 1.3–2.38, p<0.001) and married women (AOR=1.85 (95% CI: 1.39 – 2.47, p<0.001), were significantly associated with the need to access healthcare services Table 5.

For peer review only

Table 5: Bivariate and multivariate logistic regression analysis between need to access healthcare and socio-demographic factors

Variables	Bivariate analysis		Multivariate analysis		Collinearity statistics
	**COR (95% CI)**	***P-value***	**AOR (95% CI)**	***P-value***	***VIF***
**Age groups**					**1.039***
35 – 49 (Adults)	**Ref**		**Ref**		
25 – 34 (Young Adult)	1.60 (1.06 – 1.83)	**0.002***	1.76 (1.30 – 2.38)	**< 0.001***	
15 – 24 (Youth)	0.86 (0.63 – 1.68)	0.337	1.30 (0.90 – 1.88)	**0.157**	
**Educational level**					**1.014***
Tertiary	**Ref**		**Ref**		
Secondary	0.77 (0.06 – 1.00)	**0.052***	0.78 (0.60 – 1.00)	**0.056**	
Primary	0.72 (0.36 – 1.45)	**0.359**	0.71 (0.35 – 1.45)	**0.351**	
**Marital status**					**1.421***
Unmarried	**Ref**		**Ref**		
Married/cohabiting	1.74 (1.38 – 2.21)	**< 0.001***	1.85 (1.39 – 2.47)	**< 0.001***	
**Economic reliance**					**1.052**
Not reliant	**Ref**		**-**		
Reliant	1.07 (0.76 – 1.51)	**0.705**	-	**-**	
**Household income Loss**					**1.001**
No	**Ref**		**Ref**		
Yes	1.39 (0.88 – 2.20)	**0.157**	1.32 (0.83 – 2.11)	**0.243**	

Ref: Reference group COR: Crude Odds Ratio AOR: Adjusted Odds Ratio * Significant

Discussion

COVID-19 pandemic continues to be a major global public health threat, challenging the provision of healthcare services and their accessibility. The aim of this study was to investigate the association between the COVID-19 pandemic-related factors, sociodemographic factors, and the need for visiting health facilities including family planning services in women aged 15-49 in the Kinshasa province of DRC. According to our findings, one in every three women aged 15-49 needed to visit a health facility during the COVID-19 lockdown and eight in ten among them succeeded. However, findings from rural Ethiopia showed unsuccessful attempts of women trying to access health facilities during the COVID-19 lockdown with dramatic decline in family planning accessibility[16]. Our study found that, the need to seek healthcare services during COVID-19 restrictions was significantly affected among women. Supportively, many studies have reported major needs and changes in the accessibility and utilization of healthcare services because of such measures as lockdowns and stay-at-home orders[17,18,19]. This differs from findings carried out in Uganda where access to contraceptive and post-natal care remained stable during the period of the COVID 19 pandemic[20] and a study carried out in Kenya reporting that COVID -19 did not affect access to health services[21] .

Furthermore, we found variations in the reasons for visiting health facilities, with the majority visiting purposely for general healthcare services while others were seeking for family planning services. Our study found consistent indication of major decreases in the utilization of healthcare services during the COVID-19 pandemic[22] contrary to studies conducted by[21], where there was no difference in health facility access rate during COVID-19. Similarly, the reasons for women's inability to access health care, including FP services, in the DRC have been evaluated since the start of COVID-19 restrictions and we found that most women are not trying to access health services. Others did not access healthcare services due to fear of being infected with COVID-19, inability to afford healthcare, no transportation to access healthcare, government restriction on movement, closure of healthcare facilities and partner disapproval. These reasons could explain the drastic

For peer review only - http://bmjopen.bmj.com/site/about/guidelines.xhtml

drop in the accessibility and utilization of healthcare services among women during the COVID-19 pandemic in the province of Kinshasa. According to Rocca Ihenacho and Alonso[23], the population is advised not to attend hospital unless strictly necessary; this advice seems to apply to all, including healthy pregnant women and even those with complications. Therefore, the direct and indirect effects of the COVID-19 response on women specifically pregnant women are enormous.

Our data aligns with continental data results showing a higher prevalence of income lost among women especially those from African Regions due to COVID-19 related factors[24,25]. Moreover, our study highlights the need for greater consideration of economic components of women's reproductive control especially during crisis periods. As we observed, study participants economically reliant on their partner and to other external sources for the purchase of their needs during the restrictions. Previous research indicates that women who have access to their own money are associated with an increased use of FP and other SRH services[26].

Also, we observed an attitudinal change or intentions in the need for FP services due to the COVID-19 pandemic among married and unmarried women. Though not statistically significant, we found out that, most unmarried women would be very unhappy if gotten pregnant as opposed to married women. A possible reason why unmarried women wouldn't be happy if gotten pregnant could be attributed to the socio-economic impact of COVID-19. During this time of the COVID-19 pandemic, people have questions about whether or not they should get pregnant. Being pregnant is usually a happy time graced with anticipation and joyful planning. But for many women, the COVID-19 pandemic has clouded this time with fear, anxiety, and uncertainty[27]. Presently, there's limited evidence available regarding mother-to-fetus transmission, transmission during delivery, and transmission during breastfeeding. Our findings showed that, a significant proportion of unmarried women would wait for many years before putting to birth due to COVID-19 pandemic. This is in line with results by[28] who found more than a third (37.3%) of their study participants who intended to have a child prior to the pandemic, decided to abandon their intent during the quarantine,

For peer review only

due to worries regarding future economic difficulties also, the maternal and mental health implications during the COVID-19 period have also been a call for concern with many pregnant women and studies showed higher rates of depression and self-harm ideation in women assessed during the COVID-19 outbreak than women before it[29].

There are several limitations in this study. The cross-sectional design of this study limits the possibility to draw causal conclusions. Further, the use of secondary data limited control over the data collecting process like limited ability to confirm information's from participants.

The study population was women living in Kinshasa provinces. Thus, the study findings cannot apply to women living in rural areas. Studies conducted prior to COVID-19 have shown limited access and low uptake of FP services in rural communities[30]. It would have been interesting to include more representatives from rural settings and evaluate the extend of the restriction on access to FP services on this population group. Another aspect though also regarded as a key strength to the study from another perspective, plays as a limitation as well- being a phone-based interview survey (advantage with the COVID-19 context) further limited the capacity of rural women to be part of the study, given the topography and limited access to phones by majority of the rural women. Furthermore, our study cannot ascertain the long-term impact of the COVID-19 restrictions on FP it would have been interesting to have other covariates which can hinder access to services during this period and give insights of the future possible effects. According to UNFPA worldwide annual report, 'For every 3 months the lockdown continues, up to 2 million additional women may be unable to use modern contraceptives'[31]. Given the fact that many services were closed during the pandemic, this could have some psychological effects on women who wanted services especially abortion services. But our study did not take into consideration such variable. Some strengths of this study include the data collection method used in the primary survey. It was greatly adapted to suit the current context to limit infection rates for both data collectors and study beneficiaries. Also, the ODK tool kit used helps in the proper processing and aggregation of data minimising erroneous effects from the field. To further deal with bias and missing

For peer review only

data in the study, we went ahead to carry out data weighting and imputation. Having the principal investigator of the primary study as part of this study further enabled us to understand the processes and clarity of every aspect of the primary study.

Conclusion

While everyone is facing the unprecedented challenges of COVID-19, women are bearing the brunt of the economic and societal fallout of the pandemic. Effective support and intervention for women and girls during pandemic and in crisis settings is essential as this has lasting and beneficial effects on many aspects and domains of their lives. This study shows that COVID-19 pandemic affected women in Kinshasa both socially and economically with increased economic reliant rates on their partners. Most women did not attempt accessing health services and others were afraid of being infected while trying to seek for services.

Ethics statements

Patient consent for publication

An oral informed consent form was obtained from all study participants before the interview. It allowed participants to freely choose to take part in the study and the possibility to withdraw whenever they felt insecure. In addition, the most recently updated privacy policy of PMA 2020[13] does not collect personally identifiable information about individuals and further ensures the confidentiality of data.

Ethics approval

Ethical approval was received from the University of Kinshasa School of Public Health (ESP/CEI/78/2020) and the John Hopkins Bloomberg School of Public Health Institutional Review Board.

Contributors:

NFF conceptualized and drafted the manuscript. WJU and EÅ contributed to the study development and supervised the study. PAZ contributed to data and statistical screening. EEJ contributed to data screening

For peer review only

and statistical analysis. NFF and EEJ contributed to the conceptual design of the analysis. NFF and EEJ contributed to the interpretation and presentation of data. WJU,PAZ and EÅ critically revised the entire manuscript. All authors have provided intellectual content and revised the manuscript. NFF is the guarantor. The corresponding authors attests that all listed authors meet authorship criteria.

Acknowledgments

The authors would like to thank the PMA program for providing the data.

Competing interests: None declared

Funding Statement: The authors received no funding for this research work.

Data Sharing: Datasets used for the study are not publicly available and are managed very restrictively according to the ethics approval and data usage guidelines by PMA. The request for the data set should be made through the PMA data request system.

For peer review only

REFERENCES

1. World Health Organization. Overview of public health and social measures in the context of COVID-19. Interim Guide, 2020. Available: https://www.who.int/publications/i/item/overview-of-public-health-and-social-measures-in-the-context-of-covid-19 Google Scholar

2. Bolarinwa OA, Ahinkorah BO, Seidu A-A, *et al*. Mapping Evidence of Impacts of COVID-19 Outbreak on Sexual and Reproductive Health: A Scoping Review. *Healthcare* 2021; **9**:436.doi:org/10.3390/healthcare9040436https://pubmed.ncbi.nlm.nih.gov/33917784/ PubMed Google Scholar

3. Banati P, Jones N, Youssef S. Intersecting vulnerabilities: The impacts of COVID-19 on the psycho-emotional lives of young people in low- and middle-income countries. Eur. J. Dev. Res. 2020; 32:1613–1638.doi:10.1057/s41287-020-00325-5 https://pubmed.ncbi.nlm.nih.gov/33191985/ PubMed Google Scholar

4. Khan FMA, Hasan MM, Kazmi Z, *et al*. Ebola and COVID-19 in Democratic Republic of Congo: grappling with two plagues at once. Trop Med Health. 2021; 24:49(1),67. doi: 10.1186/s41182-021-00356-6 https://pubmed.ncbi.nlm.nih.gov/34429168/ PubMed

5. Simone EC, Nina G, Jérôme P, *et al.* What questions we should be asking about COVID-19 in humanitarian settings: perspectives from the Social Sciences Analysis Cell in the Democratic Republic of the Congo. *BMJ Global Health* 2020;**5**:e003607. doi: 10.1136/bmjgh-2020-003607 https://pubmed.ncbi.nlm.nih.gov/32948618/ PubMed

6. Nachega JB, Mbala-Kingebeni P, Otshudiema J, *et al.* Responding to the challenges of the dual COVID-19 and Ebola epidemics in the Democratic Republic of Congo- priorities for achieving control. *American Journal of Tropical Medicine and Hygiene* 2020;**103:**597–602. doi:10.4269/ajtmh.20-0642 https://pubmed.ncbi.nlm.nih.gov/32563272/PubMed Google Scholar

For peer review only

7. Whembolua GL, Tshiswaka DI. Public trust in the time of the coronavirus disease 2019 (COVID-19): the case of the DR Congo. *Pan African Medical Journal* 2020;**35**:2. doi:10.11604/pamj.supp.2020.35.2.22606 https://pubmed.ncbi.nlm.nih.gov/32528613/ PubMed CrossRef Google Scholar

8. UNFPA (2021). Impact of COVID-19 on Family Planning: What we know one year into the pandemic. https://www.unfpa.org/resources/impact-covid-19-family-planning-what-we-know-one-year-pandemic

9. Wood SN, Karp C, OlaOlorun F, *et al*. Need for and use of contraception by women before and during COVID-19 in four sub-Saharan African geographies: results from population-based national or regional cohort surveys. *Lancet Glob Health*. 2021;9(6):e793-e801.doi: 10.1016/S2214-109X(21)00105-4 https://pubmed.ncbi.nlm.nih.gov/34019835/ PubMed

10. Rashid SF, Theobald S, Ozano K. Towards a socially just model: balancing hunger and response to the COVID-19 pandemic in Bangladesh. *BMJ Global Health* 2020;**5**:e002715. doi: 10.1136/bmjgh-2020-002715 https://pubmed.ncbi.nlm.nih.gov/32487518/ PubMed Web of Science® Google Scholar

11. Brooks SK, Webster R, Smith LE. *et al*. The psychological impact of quarantine and how to reduce it: rapid review of the evidence. *Lancet* 2020; **395**:912–920.doi:10.1016/S0140-6736(20)30460-8 https://pubmed.ncbi.nlm.nih.gov/32112714/CrossRef Full Text Google Scholar

12. Holmes EA, O'Connor RC, Perry VH. *et al*. Multidisciplinary research priorities for the COVID-19 pandemic: a call for action for mental health science. *Lancet Psychiatry* 2020; **7**:547–560. doi:10.1016/S2215-0366(20)30168-1 https://pubmed.ncbi.nlm.nih.gov/32304649/ CrossRef Full Text Google Scholar

13. Performance Monitoring for Action. [PMA 2020]. (2020). https://www.pmadata.org/privacy-policy.

14. Hartung C, Anokwa Y, Brunette W. *et al*. Open Data Kit: Tools to Build Information services for Developing Regions. 2010. doi:10.1145/2369220.2369236 https://www.researchgate.net/publication/228637865_Open_Data_Kit_Tools_to_Build_Information_Services_for_Developing_Regions Google Scholar

For peer review only

15. Performance Monitoring for Action. [PMA 2020]. PMA COVID-19 Survey. https://www.pmadata.org/sites/default/files/2020-04/PMA-COVID-19-QRE-2020.04.28-v8-ENGLISH.pdf

16. Abdela SG, Berhanu AB, Ferede LM, *et al*. Essential Healthcare Services in the Face of COVID-19 Prevention: Experiences from a Referral Hospital in Ethiopia. *Am J Trop Med* Hyg.2020;103(3):1198-1200.doi:10.4269/ajtmh.20-0464. https://pubmed.ncbi.nlm.nih.gov/32762799/ PubMed

17. Hartnett KP, Kite-Powell A, DeVies J. National syndromic surveillance program community of practice. impact of COVID-19 pandemic on emergency department visits—United states, January 1. *MMWR Morb Mortal Wkly Rep*. 2019–May 30, 2020. 12,69 (23):699–704.doi: 10.15585/mmwr.mm6923e1 https://pubmed.ncbi.nlm.nih.gov/32525856/ PMC - PubMed

18. Baum A, Schwartz MD. Admissions to Veterans Affairs hospitals for emergency conditions during the COVID-19 pandemic. *JAMA* 2020;324:969. Doi: 10.1001/jama.2020.9972 https://pubmed.ncbi.nlm.nih.gov/32501493/ PMC - PubMed

19. Salerno R, Conti CB, De Silvestri A, et al. The impact of covid-19 pandemic on urgent endoscopy in Italy: a nation-wide multicenter study. Scand J Gastroenterol2020;55:8706.doi:10.1080/00365521.2020.1782466pmidhttp://www.ncbi.nlm.nih.gov/pubmed/32615891 PubMed

20. Burt JF , Ouma J , Lubyayi L , *et al* . Indirect effects of COVID-19 on maternal, neonatal, child, sexual and reproductive health services in Kampala, Uganda. BMJ Glob Health 2021;6:e006102. doi:10.1136/bmjgh2021006102 pmid:http://www.ncbi.nlm.nih.gov/pubmed/34452941 Abstract/FREE Full TextGoogle Scholar

21. Shikuku D, Nyaoke I, Gichuru S. Early indirect impact of COVID-19 pandemic on utilization and outcomes of reproductive, maternal, newborn, child and adolescent health services in Kenya. medRxiv

For peer review only

2020;20191247.doi:10.1101/2020.09.09.20191247 https://www.medrxiv.org/content/10.1101/2020.09.09.20191247v1 Google Scholar

22. Zhang YN, Chen Y, Wang Y, *et al*. Reduction in healthcare services during the COVID-19 pandemic in China. BMJ Glob Health. 2020;5(11):e003421. DOI: 10.1136/bmjgh-2020-003421 https://gh.bmj.com/content/6/10/e005988 PubMed CrossRef Google Scholar

23. Rocca-Ihenacho L., Alonso C. Where do women birth during a pandemic? Changing perspectives on Safe Motherhood during the COVID-19 pandemic. J. Glob. Health Sci.2020;2 (1), 1–2. 10.35500/jghs.2020.2.e4 https://doi.org/10.18332/ejm/121096 CrossRef Google Scholar

24. Luisa S, Joseph F, Cory N, *et al*. Quantifying the effects of the COVID-19 pandemic on gender equality on health, social, and economic indicators: a comprehensive review of data from March, 2020, to September,2021. *Lancet* 2022;**399**(10344):2381-2397. doi.org/10.1016/S0140-6736(22)00008-3 https://pubmed.ncbi.nlm.nih.gov/35247311/ PubMed

25. Egger D, Miguel E, Warren S, *et al*. Falling living standards during the COVID-19 crisis: Quantitative evidence from nine developing countries. *Sci. Adv.* 2021;7(6): 1–12.doi: 10.1126/sciadv.abe0997https://pubmed.ncbi.nlm.nih.gov/33547077/ PMC - PubMed

26. Elizabeth R, Balaiah D, Anindita D, *et al*. Access to Money and Relation to Women's Use of Family Planning Methods among Young Married Women in Rural India. *Maternal and Child Health Journal* 2016; 20:1203–1210. doi:10.1007/s10995-016-1921-4 https://pubmed.ncbi.nlm.nih.gov/26971270/ PMC - PubMed

27. Ravaldi C, Wilson A, Ricca V, *et al*. Pregnant women voice their concerns and birth expectations during the COVID-19 pandemic in Italy. Women Birth. 2021;34(4):335-343. doi:10.1016/j.wombi.2020.07.002 https://pubmed.ncbi.nlm.nih.gov/32684343/ PMC PubMed

For peer review only

28. Mitko M, Michal D, Clifford L. A comprehensive review of the impact of COVID-19 on human reproductive biology, assisted reproduction care and pregnancy: a Canadian perspective. Journal of Ovarian Research 2020; 13(140). 10.1186/s13048-020-00737 1. https://pubmed.ncbi.nlm.nih.gov/33246480/ PMC - PubMed

29. Vardi N, Zalsman G, Madjar N, *et al*. COVID-19 pandemic: Impacts on mothers' and infants' mental health during pregnancy and shortly thereafter. Clin Child Psychol Psychiatry. 2022;27(1):82-88. doi: 10.1177/13591045211009297 https://pubmed.ncbi.nlm.nih.gov/33855857/ PMC PubMed

30. Prata N. Making family planning accessible in resource-poor settings. Philosophical Transactions of the Royal Society B Biological Science.2009; 364(1532),3093–9. https://doi.org/10.1098/rstb.2009.0172 PMC PubMed

31. United Nations Fund for Population Activities [UNFPA]. Impact of the COVID-19 pandemic on family planning and ending gender-based violence, female genital mutilation, and child marriage. UNFPA Interim technical note. 2020

For peer review only

For peer review only

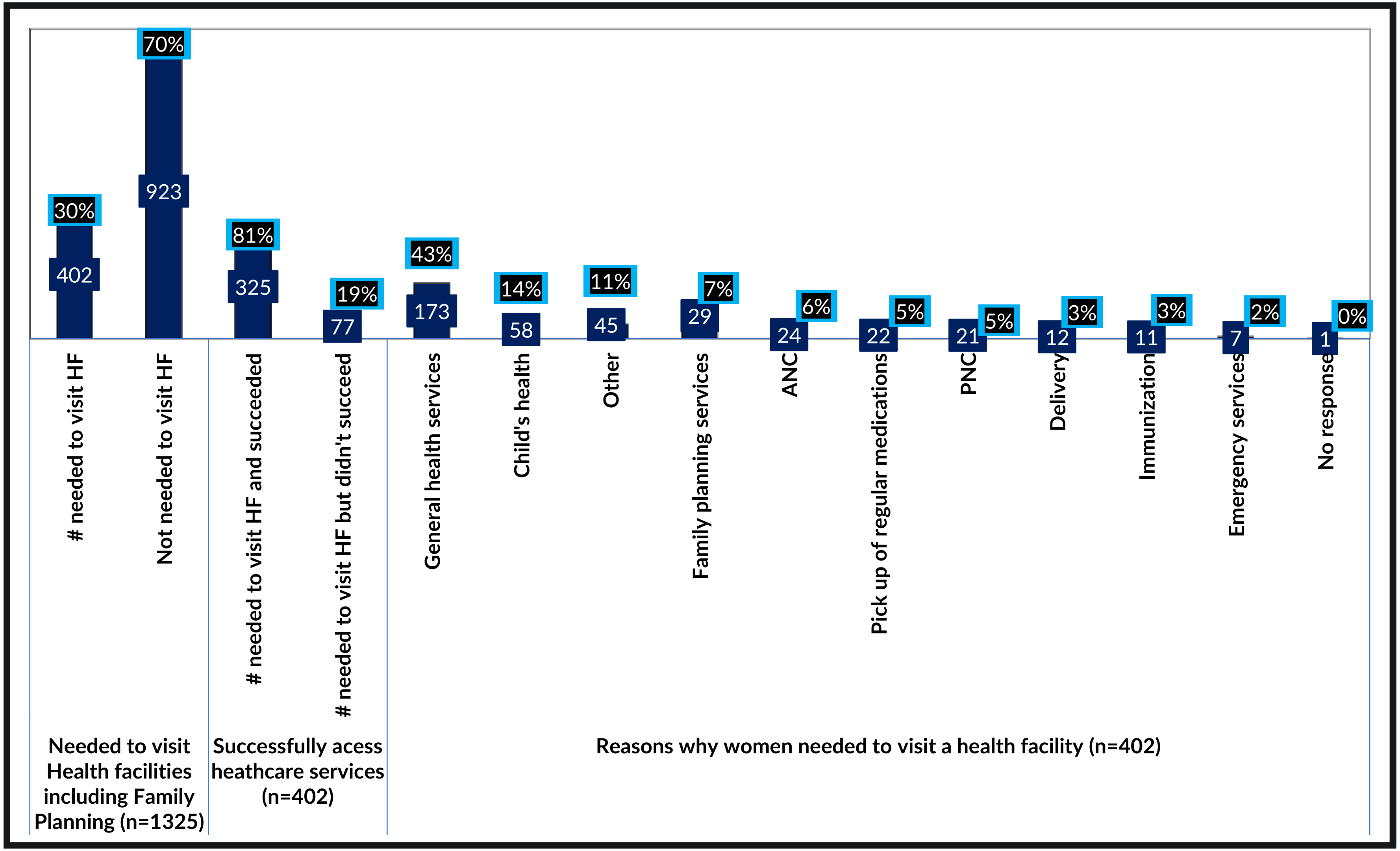

Figure 1: Need/Reasons to visit health facilities during COVID-19

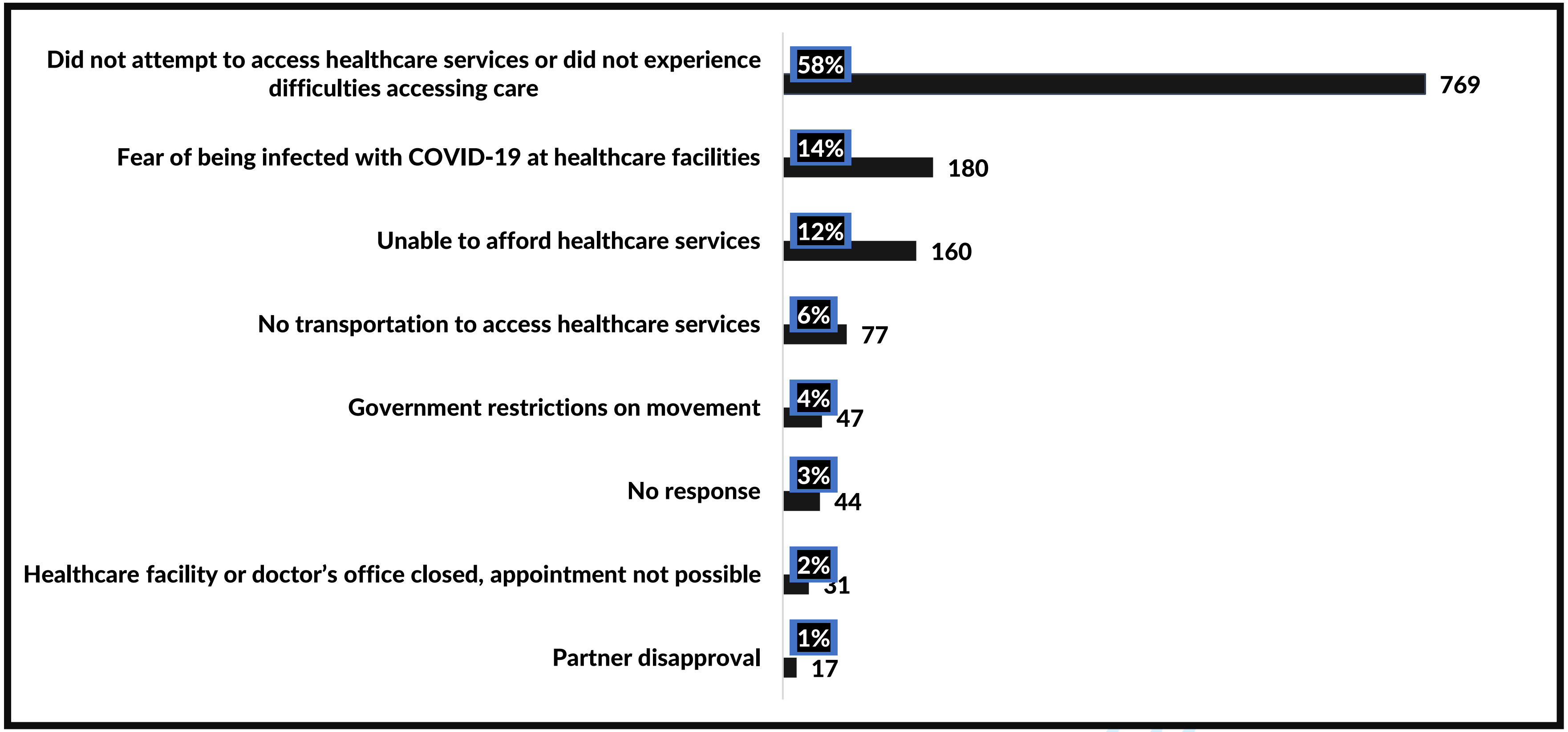

Figure 2: Primary reasons for the inaccessibility to healthcare services among women since COVID-19 restrictions (n = 1325)

Reporting checklist for cross sectional study.

Based on the STROBE cross sectional guidelines.

Instructions to authors

Complete this checklist by entering the page numbers from your manuscript where readers will find each of the items listed below.

Your article may not currently address all the items on the checklist. Please modify your text to include the missing information. If you are certain that an item does not apply, please write "n/a" and provide a short explanation.

Upload your completed checklist as an extra file when you submit to a journal.

In your methods section, say that you used the STROBE cross sectionalreporting guidelines, and cite them as:

von Elm E, Altman DG, Egger M, Pocock SJ, Gotzsche PC, Vandenbroucke JP. The Strengthening the Reporting of Observational Studies in Epidemiology (STROBE) Statement: guidelines for reporting observational studies.

For peer review only

		Reporting Item	Page Number
**Title and abstract**			
Title	#1a	Indicate the study’s design with a commonly used term in the title or the abstract	1
Abstract	#1b	Provide in the abstract an informative and balanced summary of what was done and what was found	2
**Introduction**			
Background / rationale	#2	Explain the scientific background and rationale for the investigation being reported	4
Objectives	#3	State specific objectives, including any prespecified hypotheses	4,5
**Methods**			
Study design	#4	Present key elements of study design early in the paper	5
Setting	#5	Describe the setting, locations, and relevant dates, including periods of	5

		recruitment, exposure, follow-up, and data collection	
Eligibility criteria	#6a	Give the eligibility criteria, and the sources and methods of selection of participants.	6
	#7	Clearly define all outcomes, exposures, predictors, potential confounders, and effect modifiers. Give diagnostic criteria, if applicable	6,7
Data sources / measurement	#8	For each variable of interest give sources of data and details of methods of assessment (measurement). Describe comparability of assessment methods if there is more than one group. Give information separately for for exposed and unexposed groups if applicable.	7
Bias	#9	Describe any efforts to address potential sources of bias	7
Study size	#10	Explain how the study size was arrived at	5
Quantitative variables	#11	Explain how quantitative variables were handled in the analyses. If applicable, describe which groupings were chosen, and why	7
Statistical methods	#12a	Describe all statistical methods, including those used to control for confounding	7
Statistical methods	#12b	Describe any methods used to examine subgroups and interactions	7
Statistical methods	#12c	Explain how missing data were addressed	7
Statistical methods	#12d	If applicable, describe analytical methods taking account of sampling strategy	7
Statistical methods	#12e	Describe any sensitivity analyses	NA
**Results**			
Participants	#13a	Report numbers of individuals at each stage of study—eg numbers potentially eligible, examined for eligibility, confirmed eligible, included in the study, completing follow-up, and analysed. Give information separately for for exposed and unexposed groups if applicable.	8
Participants	#13b	Give reasons for non-participation at each stage	8
Participants	#13c	Consider use of a flow diagram	12

For peer review only

Descriptive data	#14a	Give characteristics of study participants (eg demographic, clinical, social) and information on exposures and potential confounders. Give information separately for exposed and unexposed groups if applicable.	8,9
Descriptive data	#14b	Indicate number of participants with missing data for each variable of interest	16
Outcome data	#15	Report numbers of outcome events or summary measures. Give information separately for exposed and unexposed groups if applicable.	15
Main results	#16a	Give unadjusted estimates and, if applicable, confounder-adjusted estimates and their precision (eg, 95% confidence interval). Make clear which confounders were adjusted for and why they were included	16
Main results	#16b	Report category boundaries when continuous variables were categorized	16
Main results	#16c	If relevant, consider translating estimates of relative risk into absolute risk for a meaningful time period	16
Other analyses	#17	Report other analyses done—e.g., analyses of subgroups and interactions, and sensitivity analyses	29,30
**Discussion**			
Key results	#18	Summarise key results with reference to study objectives	17
Limitations	#19	Discuss limitations of the study, taking into account sources of potential bias or imprecision. Discuss both direction and magnitude of any potential bias.	21
Interpretation	#20	Give a cautious overall interpretation considering objectives, limitations, multiplicity of analyses, results from similar studies, and other relevant evidence.	19
Generalisability	#21	Discuss the generalisability (external validity) of the study results	19,20
**Other Information**			
Funding	#22	Give the source of funding and the role of the funders for the present study and, if applicable, for the original study on which the present article is based	NA

The STROBE checklist is distributed under the terms of the Creative Commons Attribution License CC-BY. This checklist was completed on 03. February 2023 using https://www.goodreports.org/, a tool made by the EQUATOR Network in collaboration with Penelope.ai

For peer review only

BMJ Open

The COVID-19 pandemic hits differently: examining its consequences for women's livelihoods and healthcare access. A cross-sectional study in Kinshasa DRC

Journal:	*BMJ Open*
Manuscript ID	bmjopen-2023-072869.R1
Article Type:	Original research
Date Submitted by the Author:	27-Jul-2023
Complete List of Authors:	Nkweleko Fankam, Falone; Dalarna University School of Health and Welfare,<br>Ugarte, Wiliam; Uppsala University, Department of Women's and Children's Health<br>Akilimali, Pierre; University of Kinshasa Faculty of Sciences, Department of Biostatistics and Epidemiology, Kinshasa School of Public Health<br>Ewane, Etah Junior; University of Buea, Department of Microbiology and Parasitology, Faculty of Science,<br>Eva, Åkerman; Karolinska Institute, Department of Women's and Children's Health
**Primary Subject Heading**:	Global health
Secondary Subject Heading:	Sexual health, Reproductive medicine, Public health
Keywords:	COVID-19, Public health < INFECTIOUS DISEASES, Reproductive medicine < GYNAECOLOGY

SCHOLARONE™
Manuscripts

I, the Submitting Author has the right to grant and does grant on behalf of all authors of the Work (as defined in the below author licence), an exclusive licence and/or a non-exclusive licence for contributions from authors who are: i) UK Crown employees; ii) where BMJ has agreed a CC-BY licence shall apply, and/or iii) in accordance with the terms applicable for US Federal Government officers or employees acting as part of their official duties; on a worldwide, perpetual, irrevocable, royalty-free basis to BMJ Publishing Group Ltd ("BMJ") its licensees and where the relevant Journal is co-owned by BMJ to the co-owners of the Journal, to publish the Work in this journal and any other BMJ products and to exploit all rights, as set out in our licence.

The Submitting Author accepts and understands that any supply made under these terms is made by BMJ to the Submitting Author unless you are acting as an employee on behalf of your employer or a postgraduate student of an affiliated institution which is paying any applicable article publishing charge ("APC") for Open Access articles. Where the Submitting Author wishes to make the Work available on an Open Access basis (and intends to pay the relevant APC), the terms of reuse of such Open Access shall be governed by a Creative Commons licence – details of these licences and which Creative Commons licence will apply to this Work are set out in our licence referred to above.

Other than as permitted in any relevant BMJ Author's Self Archiving Policies, I confirm this Work has not been accepted for publication elsewhere, is not being considered for publication elsewhere and does not duplicate material already published. I confirm all authors consent to publication of this Work and authorise the granting of this licence.

For peer review only

The COVID-19 pandemic hits differently: examining its consequences for women's livelihoods and healthcare access. A cross-sectional study in Kinshasa DRC

Nkweleko Fankam Falone[1], William J. Ugarte[2], Pierre Akilimali Z[3], Ewane Etah Junior[4], Eva Åkerman[5]

Authors Affiliation:

1. School of Health and Welfare, Dalarna University, Falun, Sweden.
2. Department of Women's and Children's Health, Uppsala University, Uppsala, Sweden.
3. Department of Biostatistics and Epidemiology, Kinshasa School of Public Health, University of Kinshasa, Kinshasa, P.O. Box 11850, Democratic Republic of the Congo.
4. Department of Microbiology and Parasitology, Faculty of Science, University of Buea, P.O. Box 63, Buea, SWR, Cameroon.
5. Department of Women's and Children's Health, Karolinska Institute, Stockholm, Sweden.

Corresponding authors: eva.akerman.2@ki.se, fankam93@yahoo.com

Disclaimers

All the views and opinions presented in this paper are those of the authors and do not represent those of any institution.

For peer review only

Abstract

Objectives: The emergence of the COVID-19 pandemic led to multiple preventive actions as primary interventions to contain the spread of the virus. Globally, countries are facing enormous challenges with consequences for use of social, economic and health services. The Democratic Republic of Congo (DRC) was among the African countries implementing strict lockdown at the start of the pandemic, resulting in shortages and decreased access to services. The adverse effects of the pandemic had unpleasant consequences for the country. This study aimed to examine the association between COVID-19 pandemic-related factors, sociodemographic factors, and the need to visit healthcare facilities, including family planning services, among women aged 15–49 years in the DRC.

Methods: We conducted a secondary analysis of a performance monitoring for action (PMA) cross-sectional COVID-19 phone survey in Kinshasa, DRC, which had a response rate of 74.7%. In total, 1,325 randomly selected women aged 15–49 years from the Kinshasa province who had previously participated in the PMA baseline survey participated in the survey. Bivariate and multivariate logistic regressions were used to assess associations.

Results: The COVID-19 pandemic and related factors affected 92% of women in the Kinshasa province socioeconomically. A majority were highly economically dependent on their partner or some other sources for their basic needs to be met, and even more worried about the future impact of the pandemic on their household finances. Over 50% of women did not attempt visiting a health service, with some of the top reasons being fear of being infected with COVID-19 and not being able to afford services. We found a significant association between age groups and contraceptive use. The need for and use of contraceptive was higher among women aged 25–34 years than those aged 15–24 or 35–49 years.

Conclusion: Effective social/economic support to women and girls during pandemics and in crises is essential as it can have lasting beneficial effects on many domains of their lives, including their ability to access health services and the contraceptives of their choice.

For peer review only

Strengths and limitations of this study:

- The study used a cross-sectional design, providing a snapshot of the situation during the COVID-19 pandemic in Kinshasa, DRC.
- The study involved a large sample of 1325 women residing in Kinshasa with good response rate (74.7%).
- Data were collected using phone surveys, which minimized the risk of infection and enabled efficient data collection.

- One limitation of this study is the lack of causal conclusions due to the cross-sectional design.
- The sample is limited to women in Kinshasa, which is urban area and limiting generalizability in rural areas.

For peer review only

Data Availability statement

The datasets used for the study are not publicly available and are managed very restrictively in accordance with the ethics approval and data usage guidelines by PMA. Any request for the dataset should be made through the PMA data request system.

Introduction.

The emergence of the COVID-19 pandemic resulted in multiple restrictive actions being taken as primary preventive interventions in most countries [1]. Globally, many countries were remarkably unprepared, being far from ready to cope with the huge increase in critically ill persons. A scoping review by Bolarinwa *et al* [2] including studies from both high income and low-middle income countries, reported the severe impact of COVID-19 on family planning (FP) services, sexual behaviour, and maternal and child health services because of limited access to the preferred choices, especially for those with FP needs. Globally, the impact of the pandemic meant that sexual and reproductive health (SRH) services and support ranging from condoms, contraceptives, HIV testing and treatment, and uninterrupted hormone treatment for transgender persons might be limited, if available at all [3]. Thus, the pandemic has been a threat to several countries, stretching health systems to their limits, especially those with preexisting crises and humanitarian conditions – as is the case in the Democratic Republic of the Congo (DRC)[4]. Thus, COVID-19 intensifies pre-existing health hardships, for instance, the DRC had undergone several disease outbreaks in the last two years, including of measles, cholera, and Ebola[4]. The most recent Ebola outbreak was considered the second largest in the world [5]and claimed thousands of lives[5]. The long-lasting Ebola epidemic, combined with insecurity and armed conflicts, has caused an inefficient and insufficient supply of SRH services, particularly for women and young people[6]. In addition, lockdowns and resources allocated to the COVID-19 response further compounded these problems by limiting access to essential SRH services among women and girls [6].

Furthermore, the first lockdown after the first COVID-19 case was discovered in Kinshasa on 10 March 2020 included a suspension of flights from COVID-19 infected countries and a movement prohibition within the country, further limiting access to services[7]and possibly obstructing the

For peer review only

supply chain and increasing shortages for those who could access services. Also, people were frustrated and devasted by severe social and economic hurdles, which led to intensified struggles among women and girls [8]. A recent publication showed an increased need for contraception among nulliparous women in the DRC during the COVID-19 pandemic[9]. No such need was observed among women who already had children.

Setting aside resources and providing equal access to essential healthcare services, including FP services, during crises and pandemics is crucial. Various health and government institutions promoted a range of measures to contain the pandemic that focused on individual behaviours, with little attention to social, economic, contextual[10], or psychological factors[11,12]. Thus, this research was aimed at better understanding the social and economic consequences of the pandemic relative to the need for health services, including FP, among women in the urban setting of the DRC context. Recognizing these factors and consequences would potentially help stakeholders strengthen their preparation, budgeting, and delivery of SRH services globally and gain a better grasp of possible short- and long-term consequences of pandemic lockdowns on women.

The study aimed to investigate if there was an association between COVID-19 pandemic-related factors, sociodemographic factors, and the need for visiting health facilities, including FP services, in women aged 15–49 years in the DRC.

Methods

Study Design, Population, and Sample Size

The study used a cross-sectional design and was conducted by phone between May and June 2020. Participants were women aged 15–49 years randomly selected from households in the Kinshasa province, who participated in the Performance Monitoring for Action (PMA) baseline survey between December 2019 and February 2020[12]. Overall, 69.5% of the baseline participants

For peer review only

consented to take part in future studies and provided a phone number. Of the 1773 eligible participants, 77.6% (n=1375) were reached and 96.3% (n=1325) of them completed the phone survey for a final response rate of 74.7% among eligible women [13]. Stata survey procedures were used to account for the sampling design and selection weights.

Patient and Public Involvement

This secondary data analysis study involves data gathered from the PMA COVID-19 phone survey in Kinshasa. Hence, there was no patient or public involvement.

Data Collection

The survey was conducted by trained women in the designated localities using Open Data Kit (ODK). This is a publicly available software application to collect and administer surveys using mobile phones. The ODK questionnaire was adapted to restrict spontaneous skipping and thereby reduce data entry errors [14]. The data collected during the study were instantly aggregated, enabling simultaneous processing, and making it possible to make corrections and adjustments in real time, while still in the field. Throughout the data collection stage, regular monitoring was performed and feedback was given to interviewers in the event of errors, missing data, or form submission problems on the central server.

Measurements

The outcome variables for the analysis were the need to access health services, including FP, and current use of contraceptives. These outcome variables were captured as: the need to visit health services, difficulties accessing health services including FP services, current contraceptive use at the time of data collection, and behaviour changes in contraceptive use. Each question had a corresponding value [15].

For peer review only

Accessing health services in times of COVID-19, the exposure variable, was assessed through seven closed-ended multiple choice questions for which only one answer was accepted. They related to: a) inability to access services because of government restrictions on movement, b) inability to afford FP services, c) fear of being infected with COVID-19 at healthcare facilities, d) not attempting to access healthcare services or not experiencing any need to do so, e) having difficulties accessing transportation to reach healthcare services, f) healthcare facility or doctor's office closed, appointment not possible, g) preferred contraceptive method unavailable.

The sociodemographic variables included age, education, place of residence, pregnancy status, marital status, economic status, and decision-making ability regarding household purchases. Economic status was based on the ability of respondents to meet their needs and those of their families or being dependent on someone else for those needs to be met. This was coded as being reliant on husband/partner, relying on oneself, relying on oneself and husband/partner, relying on someone else, or no response.

Data Analysis

Statistical analysis was done using SPSS version 26 (IBM Corp., Armonk, NY). Descriptive statistics included means and standard deviations for quantitative variables and absolute frequencies and percentages for qualitative ones. Data were checked for normality and even distribution. Pearson's chi-squared (χ^2) test was used to compare differences in proportions between groups. Both bivariate and multivariate logistic regression analyses were used. Independent variables associated with the outcomes at $p < 0.20$ in the bivariate analysis were included in the multivariate analysis. The multivariate analysis was used for potential confounders. Logistic regression models were performed on the two dependent variables: need to access healthcare services and current use of

For peer review only

contraceptives. The variance inflation factor was used to test for multicollinearity between variables (predictors) in the regression model. All statistical analyses were considered significant at $p < 0.05$.

Results

Table 1 presents the sociodemographic characteristics of the women included in the study. A total of 1325 women, with a mean age of 29.49 ± 8.97 years, consented to participate in the study. Most of the women were well-educated, with 66.8% having secondary education and about one-third (29.9%) having tertiary education. More than half (53.0%) of the women were unmarried. At the time of data collection, the majority (94.0%) of the women were not pregnant.

Table 1: Demographic characteristics of the study population, N = 1325.

**Characteristics**	**Frequency (n)**	**Percentage (%)**
Age group (years)		
15–24 (Youth)	474	35.8
25–34 (Young adult)	464	35.0
35–49 (Adult)	387	29.2
Mean age (years)	29.49 ± 8.97	
Educational level[a]		
Primary	44	3.3
Secondary	884	66.8
Tertiary	395	29.9
Marital status[a]		
Married or cohabiting	620	47.0
Unmarried	703	53.0
Currently pregnant		
No	1246	94.0
Yes	72	5.4
Unsure/No response	7	0.6

[a] ***Missing value excluded***

For peer review only

Table 2 depicts the socioeconomic impact of COVID-19 restrictions on women in the Kinshasa province. Nearly all women experienced either partial (33.0%) or complete (59.0%) loss of household income. In total, 1214 (92.0%) of women experienced household income loss due to COVID-19. Similarly, most (89.8%) of the women were worried about the impact of COVID-19 on their future household finances. In terms of food security, 36.8% of the women described having witnessed household members starving because there was not enough food to eat. The women were asked 'whether they were currently economically reliant on their husband/partner for basic needs' and 'whether they were more economically reliant on their husband/partner now than before COVID-19 restrictions began'. Less than half of the women responded to these questions. Of 612 women who did respond to the first question, 392 (64.1%) stated that they were currently economically reliant on their husband/partner for basic needs. In addition, 289 (74.0%) of the 391 women had become more economically reliant on their husband/partner than before COVID-19 restrictions began, as shown in Table 2.

Table 2: Socioeconomic impact of COVID-19 restrictions on women in Kinshasa province.

Characteristics	Impact due to COVID	
	Frequency (n)	Percentage (%)
**Household income loss**		
None	107	8.0
Partial	438	33.0
Complete	776	59.0
**Total**	**1321[a]**	**100**
**Worried about the impact of COVID-19 on future household finances**		
Yes	1177	89.8
No	133	10.2
**Total**	**1310[a]**	**100**
**Lack of food for 24 hours**		
Yes	486	36.8

No	834	63.2
**Total**	**1320[a]**	**100**
**Frequency of food inadequacy (n=486)**		
Rarely (1–2 times)	171	35.2
Sometimes (3–10 times)	239	49.2
Often (> 10 times)	75	15.4
Don't know	01	0.2
**Total**	**486**	**100**
**Currently economically reliant on husband/partner for basic needs**		
Yes	392	29.6
No	220	16.6
No response	713	53.8
**Total**	**1325**	**100**
**More economically reliant on husband/partner now than before COVID-19 restrictions began**		
Yes	289	21.8
No	102	7.7
No response/do not know	934	70.5
**Total**	**1325**	**100**

[a] ***Missing value excluded***

With regard to women's needs to visit healthcare facilities for services including FP during the COVID-19 restrictions, only 402 (30.0%) needed healthcare services. Of those, 325 (81.0%) successfully visited facilities and had access to healthcare. The reasons for accessing healthcare were determined, ranked, and presented in Figure 1 (Online supplemental appendix). The largest proportion (43.0%) of the women visited health facilities for general healthcare services, though others visited the facilities due to a child's health (14.0%) or for other personal reasons (11.0%), FP services (7.0%), or antenatal care (6.0%).

Reasons for not accessing healthcare services were evaluated and presented in Figure 2 (Online supplemental appendix). More than half (58%) of the women reported not attempting to access healthcare services or not experiencing difficulties in accessing care. However, some of the women

For peer review only

did not access healthcare for fear of being infected with COVID-19 (14.0%) and others were unable to afford healthcare services (12.0%). Other reasons included no transportation to access healthcare (6.0%), government restriction on movement (4.0%), healthcare facilities or doctors' offices closed (2.0%), and partner disapproval (1.0%).

There was a self-reported attitudinal change in the need for FP due to the COVID-19 pandemic among married or cohabiting and unmarried women, as shown in Table 3. A total of 111 women stated that they had changed their mind about getting pregnant, with no statistically significant difference between unmarried and married women. Most unmarried women (57.6%) responded that they would be very unhappy if they had become pregnant, which was not seen among married women (26.9%). Furthermore, a significant proportion of the unmarried women (62.0%) expected to wait for many years before giving birth, with a smaller proportion among married women (34.7%). In line with this, more unmarried women (61.0%) than married women (38.7%) stated that they were currently using some method to delay or avoid pregnancy, as shown in Table 3.

For peer review only

Table 3: Attitudinal changes due to COVID-19 restrictions, comparing married/cohabiting with unmarried women.				
**Characteristics**	**Categories**	**Responses**		***P value***
		**Married or cohabiting. n = 620 frequency (%)**	**Unmarried. n = 703 frequency (%)**	
**Change of mind about being pregnant due to COVID-19**	Yes (n = 111)	50 (8.0)	61 (8.7)	0.691
**Would you prefer not to have any more children?**	No more children (n = 193)	137 (22.1)	56 (8.0)	**< 0.001***
**Respondent's feelings if pregnant**	Very unhappy (n = 572)	167 (26.9)	405 (57.6)	**< 0.001***
**Currently using method to delay or avoid getting pregnant**	No (n = 669)	240 (38.7)	429 (61.0)	**< 0.001***
**Waiting time to put to birth**	Years (n = 651)	215 (34.7)	436 (62.0)	**< 0.001***

For analytical purposes, married women encompassed both those legally married and those living with partners (cohabiting).

For peer review only

Both bivariate and multivariate analyses were conducted to assess relationships between current use of contraceptives and sociodemographic factors, see Table 4. We observed that significantly more women aged 25–34 years (50.4%) were using contraceptives than those aged 15–24 years (37.1%) and 35–49 years (42.0%). A chi-squared test showed a significant association between age group and current use of contraceptives. Furthermore, current use of contraceptives increased with an increase in educational level. In other words, more women with tertiary education were currently using contraceptives than those in secondary education (41.0%) and primary education (40.9%). With regard to marital status, significantly more married women (51.3%) than unmarried women (36.1%) were using contraceptives.

Though the difference was not significant, women who experienced household income loss (43.7%) used contraceptives more often than those who did not experience household income loss (39.3%). Similarly, more women who were economically reliant on their husband/partner (51.5%) reported currently using contraceptives than those not experiencing economic reliance (50.9%). After confounders in the multivariate logistic regression, being married was significantly associated (adjusted odds ratio (AOR): 2.04 (95% confidence interval (CI): 1.56–2.67, $p < 0.001$) with current use of contraceptives. Secondary education (AOR: 0.72 (95% CI: 0.57–0.92, $p = 0.009$) also increased the likelihood of current use of contraceptives.

For peer review only

Table 4: Bivariate and multivariate logistic regression analysis of current use of contraceptives and sociodemographic factors.

Variables	Bivariate analysis		Multivariate analysis (n = 572)		
	COR (95% CI)	*P value*	AOR (95% CI)	*P value*	*VIF*
**Age group (years)**					**1.039**
15–24 (Youth)	**Ref**		**Ref**		
25–34 (Young adult)	1.72 (1.34–2.24)	**< 0.001***	1.22 (0.91–1.64)	**0.177**	
35–49 (Adult)	1.23 (0.93–1.61)	**0.149**	0.78 (0.56–1.08)	**0.136**	
**Educational level**					**1.014**
Tertiary	**Ref**		**Ref**		
Secondary	0.74 (0.58–0.94)	**0.014***	0.72 (0.57–0.92)	**0.009***	
Primary	0.73 (0.39–1.39)	**0.350**	0.70 (0.37–1.35)	**0.295**	
**Marital status**					**1.000**
Unmarried	**Ref**		**Ref**		
Married/cohabiting	1.86 (1.49–2.32)	**< 0.001***	2.04 (1.56–2.67)	**< 0.001***	
**Economic reliance**					**1.052**
Not reliant	**Ref**		**Ref**		
Reliant	1.03 (0.74–1.43)	**0.883**	-	-	
**Household income loss**					**1.001**
No	**Ref**		**Ref**		
Yes	1.20 (0.80–1.80)	**0.379**	-	-	

Ref: Reference group; COR: Crude odds ratio; CI: confidence interval; AOR: Adjusted odds ratio VIF: Variance inflation factor; * Significant factor (p <0.05).

For peer review only - http://bmjopen.bmj.com/site/about/guidelines.xhtml

To ascertain the association between sociodemographic factors and the need to access healthcare including FP, both bivariate and multivariate analyses were performed, see Table 5. The bivariate analysis showed that being aged between 25–34 years (COR = 1.60 (95% CI: 1.06–1.83), being in secondary education (COR = 0.77 (95% CI: 0.06–1.00), being married (COR = 1.74 (95% CI:1.38–2.21), and having lost household income (COR = 1.39 (95% CI:0.88–2.20) were all associated with the need to access healthcare services. However, after using the multivariate analysis, we observed that being aged 25–34 years (AOR = 1.76, 95% CI: 1.3–2.38, $p<0.001$) and being married (AOR = 1.85 (95% CI: 1.39–2.47, $p<0.001$) was significantly associated with the need to access healthcare services, see Table 5.

For peer review only

Table 5: Bivariate and multivariate logistic regression analysis between need to access healthcare and sociodemographic factors.

Variables	Bivariate analysis		Multivariate analysis		Collinearity statistics
	COR (95% CI)	*P value*	AOR (95% CI)	*P value*	*VIF*
**Age group (year)**					**1.039***
35–49 (Adult)	**Ref**		**Ref**		
25–34 (Young adult)	1.60 (1.06–1.83)	**0.002***	1.76 (1.30–2.38)	**< 0.001***	
15–24 (Youth)	0.86 (0.63–1.68)	0.337	1.30 (0.90–1.88)	**0.157**	
**Educational level**					**1.014***
Tertiary	**Ref**		**Ref**		
Secondary	0.77 (0.06–1.00)	**0.052***	0.78 (0.60–1.00)	**0.056**	
Primary	0.72 (0.36–1.45)	**0.359**	0.71 (0.35–1.45)	**0.351**	
**Marital status**					**1.421***
Unmarried	**Ref**		**Ref**		
Married/cohabiting	1.74 (1.38–2.21)	**< 0.001***	1.85 (1.39–2.47)	**< 0.001***	
**Economic reliance**					**1.052**
Not reliant	**Ref**		**-**		
Reliant	1.07 (0.76–1.51)	**0.705**	-	**-**	
**Household income loss**					**1.001**
No	**Ref**		**Ref**		
Yes	1.39 (0.88–2.20)	**0.157**	1.32 (0.83–2.11)	**0.243**	

COR: Crude odds ratio CI: confidence interval AOR: Adjusted odds ratio VIF: Variance inflation factor; Ref: Reference group; * Significant factor.

Discussion

The COVID-19 pandemic continues to be a major global public health threat, challenging the provision and accessibility of healthcare services. The aim of this study was to investigate associations between COVID-19 pandemic-related factors, sociodemographic factors, and the need for visiting health facilities including FP services in women aged 15–49 years in the Kinshasa province of DRC. According to our findings, one in three women aged 15–49 years needed to visit a health facility during the COVID-19 lockdown and eight in ten among them succeeded. However, findings from rural Ethiopia showed unsuccessful attempts of women trying to access health facilities during the COVID-19 lockdown, with a dramatic decline in FP accessibility [16]. Our study found that the use of healthcare services during COVID-19 restrictions was significantly affected among women. This is in line with many studies that have reported major changes in the accessibility and utilization of healthcare services because of measures such as lockdowns and stay-at-home orders [17,18,19]. This differs from findings carried out in Uganda, where access to contraceptive and post-natal care remained stable during the COVID-19 pandemic[20] and in a study carried out in Kenya reporting that COVID-19 did not affect access to health services[21].

Furthermore, we found a range of reasons for visiting health facilities, with the majority visiting for general healthcare services and some seeking FP services. Our study found consistent indications of major decreases in the utilization of healthcare services during the COVID-19 pandemic[22], contrary to another study[21] where there was no difference in health facility access rates during the pandemic. The reasons for women's inability to access health care, including FP services, in the DRC have been evaluated after COVID-19 restrictions were implemented. We found that most women did not try to access health services. Others avoided healthcare services due to fear of being infected with COVID-19, inability to afford healthcare, not having transportation to access healthcare, government restrictions on

For peer review only - http://bmjopen.bmj.com/site/about/guidelines.xhtml

movement, closure of healthcare facilities, or partner disapproval. These reasons could explain the drastic drop in the accessibility and utilization of healthcare services among women during the COVID-19 pandemic in the province of Kinshasa. According to Rocca Ihenacho and Alonso [23], the population was advised not to visit a hospital unless strictly necessary; this advice seems to have applied to all citizens, including healthy pregnant women and even those with complications. Thus, the direct and indirect effects of the COVID-19 response on women, especially pregnant women, would be enormous.

Our data align with other results showing a higher prevalence of income lost among women, especially in African regions, due to COVID-19-related factors[24,25]. Moreover, our study highlights the need for greater consideration of economic components of women's reproductive control, particularly during crisis periods. As we observed, many study participants were economically reliant on their partner or other external sources during the period of restrictions. Previous research indicates that women who have access to their own money use more FP and other SRH services [26].

Also, we observed an attitudinal change towards FP services and altered intentions in use of such services among married and unmarried women due to the COVID-19 pandemic. Though the difference was not statistically significant, we found that more unmarried women than married women would have been very unhappy if they had gotten pregnant. A possible reason why unmarried women would not be happy if they had gotten pregnant could be the socioeconomic impact of COVID-19. During the COVID-19 pandemic, people were unsure whether or not they should get pregnant. Being pregnant is usually a happy time of anticipation and joyful planning. However, for many women, the COVID-19 pandemic clouded this period with fear, anxiety, and uncertainty [27]. Presently, there is limited evidence available regarding mother-to-foetus transmission, transmission during delivery, and transmission during breastfeeding. Our findings showed that a significant proportion of unmarried women expected to wait for many years before giving birth due to the COVID-19 pandemic. This is in line with a study [28]who

For peer review only

that found more than a third (37.3%) of their study participants who had intended to have a child prior to the pandemic changed their mind during the pandemic due to worries regarding future economic difficulties. The maternal and mental health implications during the COVID-19 pandemic have also been a for concern among many pregnant women and studies showed higher rates of depression and self-harm ideation in women assessed during the COVID-19 outbreak than before[29].

There are several limitations in this study. The cross-sectional design of this study limits the possibility to draw causal conclusions. Further, the use of secondary data limits control over the data collecting process and the ability to confirm information from participants.

The study population included only women living in the Kinshasa province. Thus, the study findings cannot be generalized to women living in rural areas. Studies conducted before the COVID-19 pandemic have shown limited access and low uptake of FP services in rural communities [30]. It would have been interesting to include more representatives from rural settings and evaluate the effects of the restrictions on access to FP services in this group. Another aspect which can be regarded as a key strength, or a limitation is that the interview survey was phone-based (which was necessary given the COVID-19 context). This further limited the ability of women in rural areas to be part of the study, due to the topography and limited access to phones among this group. Furthermore, our study cannot ascertain the long-term impact of the COVID-19 restrictions on FP. It would have been interesting to have other covariates which could have hindered access to services during the period in question and provide insights into possible future effects. As stated in the United Nations Population Fund's worldwide annual report: 'For every 3 months the lockdown continues, up to 2 million additional women may be unable to use modern contraceptives.'[31]Given the fact that many services were closed during the pandemic, this could have some psychological effects on women who wanted servics, especially abortion services. However, our study did not take any such variable into consideration. Some strengths of this study include

For peer review only

the data collection method used in the primary survey. It was well-adapted to suit the current context and to limit infection rates for both data collectors and study participants. Also, the ODK tool kit aided proper processing and aggregation of data, minimising errors in the field. To further deal with bias and missing data in the study, we performed data weighting and imputation. Having the principal investigator of the primary study as part of this study further enabled us to understand the processes and gain clarity on every aspect of the primary study.

Conclusion

While everyone has faced and is facing the unprecedented challenges of COVID-19, women are bearing the brunt of the economic and societal fallout of the pandemic. Effective support and interventions for women and girls during pandemics and crises are essential as they can have lasting beneficial effects on many aspects and domains of life. This study shows that the COVID-19 pandemic affected women in Kinshasa both socially and economically, with increased rates of economic reliance on a partner. Considering the potential disruptions to contraceptive services, healthcare providers could engage in discussions with women about self-administered subcutaneous depot shots or explore the use of long-term methods like intrauterine devices (IUDs) to ensure consistent and reliable contraception. Most women did not attempt to access health services and some were afraid of being infected if they were to seek services.

Ethics statements

Patient consent for publication.

Verbal informed consent was obtained from each study participant before their interview. It was stated that participants could freely choose to take part in the study and had the possibility to withdraw at any time if they so desired. In addition, in accordance with the most recently updated privacy policy of PMA,

For peer review only

from 2020[13], personally identifiable information about individuals are not collected and data confidentiality is protected.

Ethics approval

Ethics approval was received from the University of Kinshasa School of Public Health (ESP/CEI/78/2020) and the John Hopkins Bloomberg School of Public Health Institutional Review Board.

Contributors:

NFF conceptualized and drafted the manuscript. WJU and EÅ contributed to the study development and supervised the study. PAZ contributed to data and statistical screening. EEJ contributed to data screening and statistical analysis. NFF and EEJ contributed to the conceptual design of the analysis. NFF and EEJ contributed to the interpretation and presentation of data. WJU, PAZ and EÅ critically revised the entire manuscript. All authors have provided intellectual content and revised the manuscript. NFF is the guarantor. The corresponding authors attest that all listed authors meet authorship criteria.

Acknowledgments

The authors would like to thank the PMA programme for providing the data.

Competing interests: None declared

Funding Statement: The authors received no funding for this research work.

Data Sharing: Datasets used for the study are not publicly available and are managed very restrictively in accordance with the ethics approval and data usage guidelines by PMA. Any request for the dataset should be made through the PMA data request system.

For peer review only

Figure 1: Need/Reasons to visit health facilities during COVID-19

Figure 2: Primary reasons for the inaccessibility to healthcare services among women since COVID-19 restrictions (n = 1325)

For peer review only

REFERENCES

1. World Health Organization. Overview of public health and social measures in the context of COVID-19. Interim Guide, 2020. Available: https://www.who.int/publications/i/item/overview-of-public-health-and-social-measures-in-the-context-of-covid-19
2. Bolarinwa OA, Ahinkorah BO, Seidu A-A, et al. Mapping evidence of impacts of COVID-19 outbreak on sexual and reproductive health: a scoping review. Healthc. 2021; 9:436. doi:10.3390/healthcare9040436
3. Banati P, Jones N, Youssef S. Intersecting vulnerabilities: The impacts of COVID-19 on the psycho-emotional lives of young people in low- and middle-income countries. Eur J Dev Res. 2020; 32:1613-38. doi:10.1057/s41287-020-00325-5
4. Khan FMA, Hasan MM, Kazmi Z, *et al*. Ebola and COVID-19 in Democratic Republic of Congo: grappling with two plagues at once. Trop Med Health. 2021;24:49(1),67. doi:10.1186/s41182-021-00356-6
5. Carter SE, Gobat N, Pfaffmann Zambruni J, et al. What questions we should be asking about COVID-19 in humanitarian settings: perspectives from the Social Sciences Analysis Cell in the Democratic Republic of the Congo. BMJ Global Health. 2020;5:e003607. doi:10.1136/bmjgh-2020-003607
6. Nachega JB, Mbala-Kingebeni P, Otshudiema J, et al. Responding to the challenges of the dual COVID-19 and Ebola epidemics in the Democratic Republic of Congo- priorities for achieving control. Am J Trop Med Hyg. 2020;103:597-602. doi:10.4269/ajtmh.20-0642
7. Whembolua GL, Tshiswaka DI. Public trust in the time of the coronavirus disease 2019 (COVID-19): the case of the DR Congo. Pan African Medical Journal. 2020;35:2. doi:10.11604/pamj.supp.2020.35.2.22606
8. UNFPA (2021). Impact of COVID-19 on family planning: What we know one year into the pandemic. https://www.unfpa.org/resources/impact-covid-19-family-planning-what-we-know-one-year-pandemic
9. Wood SN, Karp C, OlaOlorun F, et al. Need for and use of contraception by women before and during COVID-19 in four sub-Saharan African geographies: results from population-

For peer review only

based national or regional cohort surveys. Lancet Glob Health. 2021;9(6):e793-e801. doi:10.1016/S2214-109X(21)00105-4

10. Rashid SF, Theobald S, Ozano K. Towards a socially just model: balancing hunger and response to the COVID-19 pandemic in Bangladesh. BMJ Global Health. 2020;5:e002715. doi:10.1136/bmjgh-2020-002715
11. Brooks SK, Webster R, Smith LE, et al. The psychological impact of quarantine and how to reduce it: rapid review of the evidence. Lancet. 2020;395:912-20. doi:10.1016/S0140-6736(20)30460-8
12. Holmes EA, O'Connor RC, Perry VH, et al. Multidisciplinary research priorities for the COVID-19 pandemic: a call for action for mental health science. Lancet Psychiatry. 2020;**7**:547-60. doi:10.1016/S2215-0366(20)30168-1
13. Performance Monitoring for Action. [PMA 2020]. (2020). https://www.pmadata.org/privacy-policy.
14. Hartung C, Anokwa Y, Brunette W, et al. Open Data Kit: tools to build information services for developing regions. 2010. ICTD '10: Proceedings of the 4th ACM/IEEE International Conference on Information and Communication Technologies and Development. Article No. 18, pages 1-12. doi:10.1145/2369220.2369236
15. Performance Monitoring for Action. [PMA 2020]. PMA COVID-19 Survey. https://www.pmadata.org/sites/default/files/2020-04/PMA-COVID-19-QRE-2020.04.28-v8-ENGLISH.pdf
16. Abdela SG, Berhanu AB, Ferede LM, et al. Essential healthcare services in the face of COVID-19 prevention: experiences from a referral hospital in Ethiopia. Am J Trop Med Hyg. 2020;103(3):1198-200. doi:10.4269/ajtmh.20-0464
17. Hartnett KP, Kite-Powell A, DeVies J. National syndromic surveillance program community of practice. impact of COVID-19 pandemic on emergency department visits—United States, January 1. *MMWR Morb Mortal Wkly Rep*. 2019–May 30, 2020. 12,69 (23):699-704. doi:10.15585/mmwr.mm6923e1

For peer review only

18. Baum A, Schwartz MD. Admissions to Veterans Affairs hospitals for emergency conditions during the COVID-19 pandemic. JAMA. 2020;324:969. doi:10.1001/jama.2020.9972

19. Salerno R, Conti CB, De Silvestri A, et al. The impact of covid-19 pandemic on urgent endoscopy in Italy: a nation-wide multicenter study. Scand J Gastroenterol. 2020;55:8706. doi:10.1080/00365521.2020.1782466

20. Burt JF, Ouma J, Lubyayi L, et al. Indirect effects of COVID-19 on maternal, neonatal, child, sexual and reproductive health services in Kampala, Uganda. BMJ Glob Health. 2021;6:e006102. doi:10.1136/bmjgh2021006102

21. Shikuku D, Nyaoke I, Gichuru S. Early indirect impact of COVID-19 pandemic on utilization and outcomes of reproductive, maternal, newborn, child and adolescent health services in Kenya. medRxiv. 2020; 20191247. doi:10.1101/2020.09.09.20191247

22. Zhang YN, Chen Y, Wang Y, et al. Reduction in healthcare services during the COVID-19 pandemic in China. BMJ Glob Health. 2020;5(11):e003421. doi:10.1136/bmjgh-2020-003421

23. Rocca-Ihenacho L, Alonso C. Where do women birth during a pandemic? Changing perspectives on Safe Motherhood during the COVID-19 pandemic. J Glob Health Sci. 2020;2(1), 1-2. 10.35500/jghs.2020.2.e4 doi:10.18332/ejm/121096

24. Luisa S, Joseph F, Cory N, et al. Quantifying the effects of the COVID-19 pandemic on gender equality on health, social, and economic indicators: a comprehensive review of data from March, 2020, to September, 2021. Lancet 2022;399(10344):2381-97. doi: 10.1016/S0140-6736(22)00008-3

For peer review only

25. Egger D, Miguel E, Warren S, et al. Falling living standards during the COVID-19 crisis: quantitative evidence from nine developing countries. Sci Adv. 2021;7(6):1-12. doi:10.1126/sciadv.abe0997

26. Reed E, Donta B, Dasgupta A, et al. Access to money and relation to women's use of family planning methods among young married women in rural India. Maternal and Child Health Journal. 2016;20:1203-10. doi:10.1007/s10995-016-1921-4

27. Ravaldi C, Wilson A, Ricca V, et al. Pregnant women voice their concerns and birth expectations during the COVID-19 pandemic in Italy. Women Birth. 2021;34(4):335-43. doi:10.1016/j.wombi.2020.07.002

28. Mitko M, Michal D, Clifford L. A comprehensive review of the impact of COVID-19 on human reproductive biology, assisted reproduction care and pregnancy: a Canadian perspective. Journal of Ovarian Research 2020;13(140). doi:10.1186/s13048-020-00737 1.

29. Vardi N, Zalsman G, Madjar N, et al. COVID-19 pandemic: Impacts on mothers' and infants' mental health during pregnancy and shortly thereafter. Clin Child Psychol Psychiatry. 2022;27(1):82-88. doi:10.1177/13591045211009297

30. Prata N. Making family planning accessible in resource-poor settings. Philosophical Transactions of the Royal Society B Biological Science. 2009;364(1532):3093-9. doi:10.1098/rstb.2009.0172

31. United Nations Fund for Population Activities [UNFPA]. Impact of the COVID-19 pandemic on family planning and ending gender-based violence, female genital mutilation, and child marriage. UNFPA Interim Technical Note. 2020.

For peer review only

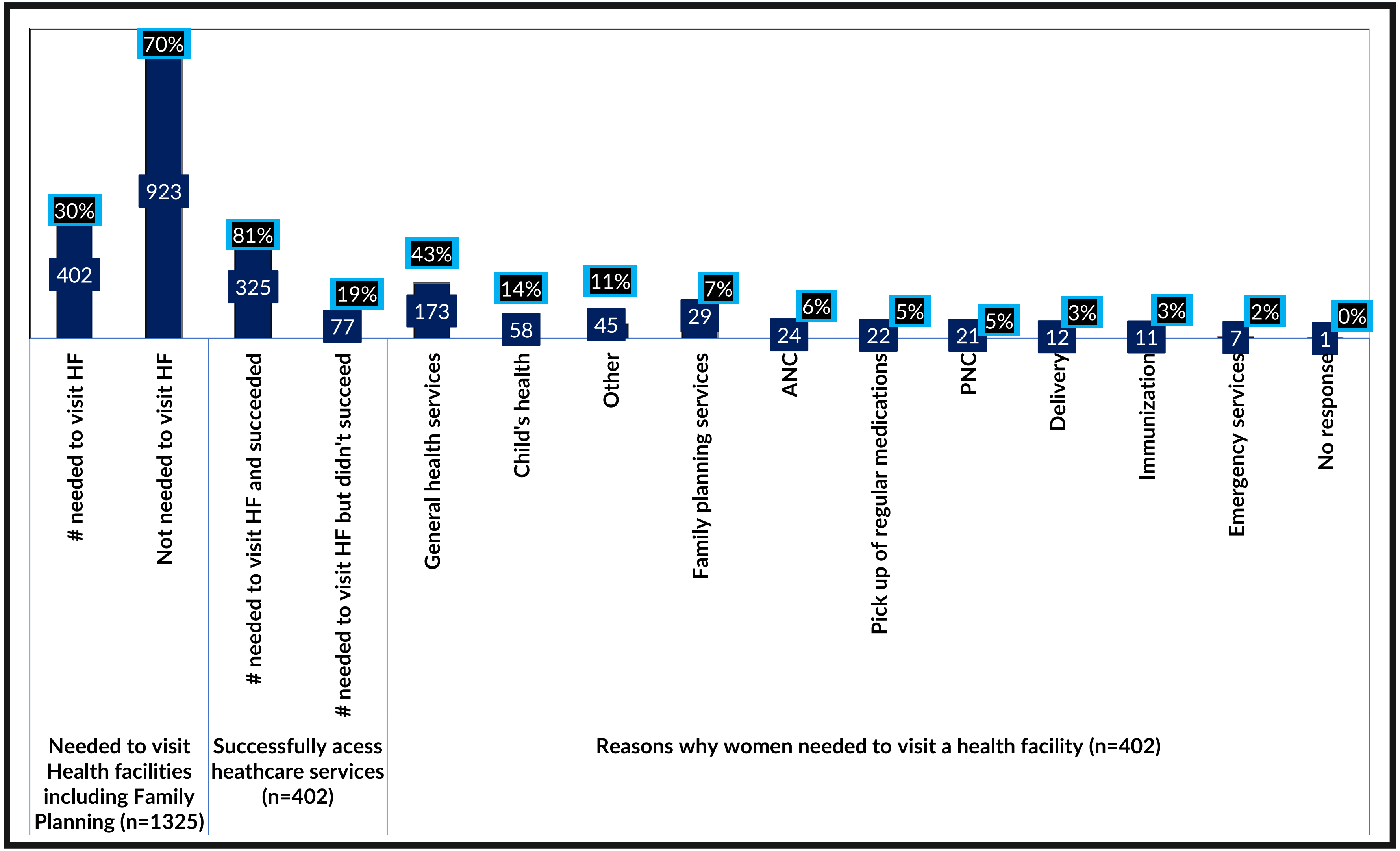

Figure 1: Need/Reasons to visit health facilities during COVID-19

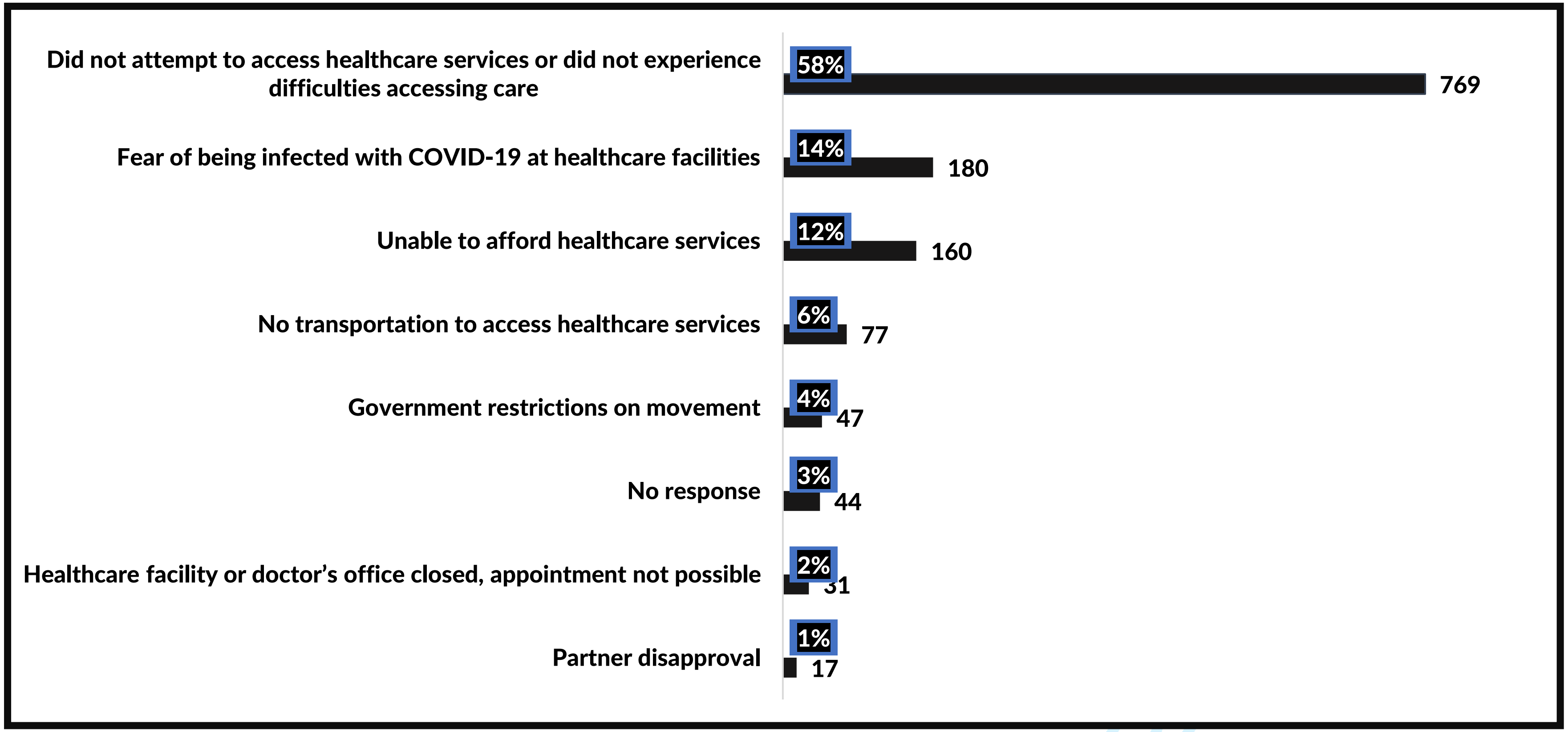

Figure 2: Primary reasons for the inaccessibility to healthcare services among women since COVID-19 restrictions (n = 1325)

Reporting checklist for cross sectional study.

Based on the STROBE cross sectional guidelines.

Instructions to authors

Complete this checklist by entering the page numbers from your manuscript where readers will find each of the items listed below.

Your article may not currently address all the items on the checklist. Please modify your text to include the missing information. If you are certain that an item does not apply, please write "n/a" and provide a short explanation.

Upload your completed checklist as an extra file when you submit to a journal.

In your methods section, say that you used the STROBE cross sectionalreporting guidelines, and cite them as:

von Elm E, Altman DG, Egger M, Pocock SJ, Gotzsche PC, Vandenbroucke JP. The Strengthening the Reporting of Observational Studies in Epidemiology (STROBE) Statement: guidelines for reporting observational studies.

		Reporting Item	Page Number
**Title and abstract**			
Title	#1a	Indicate the study’s design with a commonly used term in the title or the abstract	1
Abstract	#1b	Provide in the abstract an informative and balanced summary of what was done and what was found	2
**Introduction**			
Background / rationale	#2	Explain the scientific background and rationale for the investigation being reported	4
Objectives	#3	State specific objectives, including any prespecified hypotheses	4,5
**Methods**			
Study design	#4	Present key elements of study design early in the paper	5
Setting	#5	Describe the setting, locations, and relevant dates, including periods of	5

For peer review only

		recruitment, exposure, follow-up, and data collection	
Eligibility criteria	#6a	Give the eligibility criteria, and the sources and methods of selection of participants.	6
	#7	Clearly define all outcomes, exposures, predictors, potential confounders, and effect modifiers. Give diagnostic criteria, if applicable	6,7
Data sources / measurement	#8	For each variable of interest give sources of data and details of methods of assessment (measurement). Describe comparability of assessment methods if there is more than one group. Give information separately for for exposed and unexposed groups if applicable.	7
Bias	#9	Describe any efforts to address potential sources of bias	7
Study size	#10	Explain how the study size was arrived at	5
Quantitative variables	#11	Explain how quantitative variables were handled in the analyses. If applicable, describe which groupings were chosen, and why	7
Statistical methods	#12a	Describe all statistical methods, including those used to control for confounding	7
Statistical methods	#12b	Describe any methods used to examine subgroups and interactions	7
Statistical methods	#12c	Explain how missing data were addressed	7
Statistical methods	#12d	If applicable, describe analytical methods taking account of sampling strategy	7
Statistical methods	#12e	Describe any sensitivity analyses	NA
**Results**			
Participants	#13a	Report numbers of individuals at each stage of study—eg numbers potentially eligible, examined for eligibility, confirmed eligible, included in the study, completing follow-up, and analysed. Give information separately for for exposed and unexposed groups if applicable.	8
Participants	#13b	Give reasons for non-participation at each stage	8
Participants	#13c	Consider use of a flow diagram	12

For peer review only

Descriptive data	#14a	Give characteristics of study participants (eg demographic, clinical, social) and information on exposures and potential confounders. Give information separately for exposed and unexposed groups if applicable.	8,9
Descriptive data	#14b	Indicate number of participants with missing data for each variable of interest	16
Outcome data	#15	Report numbers of outcome events or summary measures. Give information separately for exposed and unexposed groups if applicable.	15
Main results	#16a	Give unadjusted estimates and, if applicable, confounder-adjusted estimates and their precision (eg, 95% confidence interval). Make clear which confounders were adjusted for and why they were included	16
Main results	#16b	Report category boundaries when continuous variables were categorized	16
Main results	#16c	If relevant, consider translating estimates of relative risk into absolute risk for a meaningful time period	16
Other analyses	#17	Report other analyses done—e.g., analyses of subgroups and interactions, and sensitivity analyses	29,30
**Discussion**			
Key results	#18	Summarise key results with reference to study objectives	17
Limitations	#19	Discuss limitations of the study, taking into account sources of potential bias or imprecision. Discuss both direction and magnitude of any potential bias.	21
Interpretation	#20	Give a cautious overall interpretation considering objectives, limitations, multiplicity of analyses, results from similar studies, and other relevant evidence.	19
Generalisability	#21	Discuss the generalisability (external validity) of the study results	19,20
**Other Information**			
Funding	#22	Give the source of funding and the role of the funders for the present study and, if applicable, for the original study on which the present article is based	NA

The STROBE checklist is distributed under the terms of the Creative Commons Attribution License CC-BY. This checklist was completed on 03. February 2023 using https://www.goodreports.org/, a tool made by the EQUATOR Network in collaboration with Penelope.ai

For peer review only
